# Implications of a Carbon Tax Mechanism in Remanufacturing Outsourcing on Carbon Neutrality

**DOI:** 10.3390/ijerph19095520

**Published:** 2022-05-02

**Authors:** Jie Deng, Xuwei Luo, Mengsi Hu

**Affiliations:** 1 Law School, Southwestern University of Finance and Economics, Chengdu 610074, China; dengjie121@smail.swufe.edu.cn; 2 School of Management and Economic, University of Electronic Science and Technology of China, Chengdu 611731, China; 202032150623@std.uestc.edu.cn

**Keywords:** sustainable operations, carbon monitoring, environmental policy, remanufacturing

## Abstract

Many governments have imposed methods such as a carbon tax that aim to even out the negative effects of carbon emissions. The taxes levied on different agents lead to different make–buy decisions for production structures and different environmental outcomes. Some original equipment manufacturers (OEMs) outsource remanufacturing to independent remanufacturers (IRs). Thus, a question arises: What are the implications of carbon taxes levied on different agents on remanufacturing outsourcing decisions? To answer this question, we developed two models: (1) acting as common brand owners, OEMs can be taxed for both new and remanufactured products, or (2) acting as different emitters for production and remanufacturing, OEMs are taxed for new products; however, all carbon taxes related to remanufacturing are levied on IRs. Our analysis reveals that, regarding economic performance, firms should undertake a carbon emission tax on their own initiative because this allows the taxpayer to choose more units for its preferred products and leaves its rivals at a huge disadvantage. Moreover, regarding environmental sustainability, carbon emission taxes indeed lead to mitigating the effects of carbon emissions per unit; however, environmental agencies should also pay attention to reducing the total carbon emissions by limiting the volume effects.

## 1. Introduction

The traditional industrial structure has led to an increase in greenhouse gas emissions that endangers our daily lives. Thus, governments and institutions have recognized the important roles they play in coping with climate change [1,2]. For example, the United States announced that by 2025, they will reduce their carbon emissions to 26–28% below the levels in 2005 [3]. Similarly, the Chinese government stated that China’s carbon emissions will peak before 2030 and they will achieve carbon neutrality by 2060 [4]. Similar efforts on carbon reduction can be seen in other countries, such as the European Union, Switzerland, Ireland, Denmark, and Finland, etc.

Accordingly, to reduce their carbon emissions, more and more governments and institutions have developed and/or set policies, laws, and other support mechanisms that can decouple growth from finite resource consumption and environmental degradation. For example, some governments such as those of Switzerland, Ireland, Denmark, and Finland, have imposed necessary methods, such as technology standards, emission trading systems, deposit systems, carbon taxes, and so forth, to limit carbon emissions and consumption. Among these methods, a carbon tax is highly recommended because it is a cost-effective instrument for achieving a given abatement target [5,6].

The efforts in carbon reduction naturally impact decisions on production structures, environmental tax payments, and recycling decisions [7,8]. For example, to deal with a possible carbon tax burden, some original equipment manufacturers (OEMs) use technological advancements to implement green practices as a key characteristic in their existing business systems. In recent decades, in the automobile industry, many OEMs, such as Tesla Motors, General Motors, and Ford, have provided electric vehicles with a power electronics module that is more environmentally friendly than the other models using standard motor oils [9]. Recently, low carbon technology has begun to be a key driver of the circular economy, resulting in less carbon production [10,11].

On the other hand, alongside the technological advancement to be green, more and more OEMs have developed remanufacturing as an integral part of their existing business systems due to its more environmentally friendly production [12]. Although the infrastructure for collecting used products and/or remanufacturing expertise are lacking, some OEMs have outsourced their remanufacturing operations to independent remanufacturers (IRs) [13,14]. For example, Land Rover and Caterpillar have signed a memorandum of understanding that allows Caterpillar Remanufacturing Services to act as its lead global remanufacturing services provider [15]. Similarly, IBM has outsourced its remanufacturing operations to many selected independent remanufacturers. According to a survey of the US remanufacturing industry, IRs account for 94% of the firms that engage in remanufacturing in the US market [16]. 

It should be noted that the outsourcing of remanufacturing involves different stakeholders. Although OEMs outsource remanufacturing operations to IRs, as with new products, they still control the brands for the remanufactured products. For example, IBM created certification programs for its IRs, where all remanufactured products will be inspected by IBM engineers. As a result, IBM not only has extended producer responsibility for all remanufactured equipment but also obtains a substantial relicensing fee for remanufacturing outsourcing. Therefore, although OEMs have outsourced their remanufacturing, acting as common brand owners, OEMs are still the ideal target for carbon emission policies [6,17,18]. In addition, along with outsourcing remanufacturing operations, OEMs, e.g., IBM, allow IRs to make their remanufactured products and leave an additional marginal profit for them. Therefore, acting as the actual emitters for production and remanufacturing, the OEM and/or IR should also be responsible for carbon regulations for production and/or remanufacturing [6,18]. Thus, remanufacturing outsourcing is not related to different operation decision makers but creates different taxpayers for carbon taxes. 

From a research perspective, a fundamental question arises: What are the implications of carbon taxes on remanufacturing outsourcing decisions? The answer is not obvious from the current literature, particularly in spite the fact that a number of analyses have highlighted the roles of different agents regarding remanufacturing outsourcing (see, e.g., Zhang et al. [14], Chai et al. [19], and Huang et al. [20]), and little attention has been paid to how carbon taxes levied on different agents impact the decisions of remanufacturing outsourcing. Conversely, although several studies have addressed the implications of carbon taxes set for different agents (see, e.g., Chung et al. [21] and Joan et al. [22]), remanufacturing outsourcing has not been addressed. As such, to fill this gap, we intend to highlight the implications of carbon taxes on remanufacturing outsourcing decisions as follows:

1. How do carbon taxes levied on different agents impact the decisions of remanufacturing outsourcing?
2. Which taxpayer is more beneficial for economic performance?
3. Which taxpayer is more beneficial for environmental sustainability?

More specifically, we developed two models related to remanufacturing outsourcing with two possible options for carbon taxes levied on different agents: (1) acting as common brand owners, OEMs can be taxed for both new and remanufactured products (we refer to Model C as the situation where carbon taxes related to remanufacturing are levied on the common brand owners of OEMs), or (2) acting as different emitters for production and remanufacturing, OEMs are taxed only for new products; however, all carbon taxes related to remanufacturing are levied on IRs (we refer to Model I as the situation where carbon taxes related to remanufacturing are levied on IRs). Using these two theoretical models, our analysis revealed that, from an economic perspective, carbon taxpayers usually choose more units of their preferred products to offset the negative effects of cannibalization problems. Moreover, from an environmental sustainability angle, our analysis indicates that carbon emission taxes can lead to mitigating the effects of the carbon emissions per unit; however, environmental groups and agencies should also pay attention to the mitigation effects on the total carbon emissions with volume effects.

The remainder of this paper is organized as follows. Section 2 explains our contributions by reviewing the related literature. Section 3 describes both models. Section 4 provides an analysis comparing both models. Section 5 presents the conclusion.

## 2. Literature Review

This paper is related to several streams of the literature: (1) governance on carbon emissions; (2) remanufacturing outsourcing; (3) carbon taxes levied on different agents; and (4) sustainability in remanufacturing (a quick overview can be found in Table 1).

The first stream highlights governance on carbon emissions. Wang et al. [23] characterized the optimal carbon emission tax policy through a two-period production decision model. Similarly, Zhou et al. [24] determined the optimal acquisition and remanufacturing policies of independent remanufacturing systems, where the remanufacturing time is defined by the quality of the acquired cores. Liu et al. [25] determine the remanufacturing quantity that maximizes the total profits under three common carbon emission regulation policies, such as a mandatory carbon emissions capacity, carbon tax, and cap and trade. Dou et al. [18] modeled a manufacturer who produces new products in the first period and makes new and remanufactured products in the second period under carbon tax regulations. Chai et al. [26] developed a model to derive the favorable conditions under which a carbon cap and trade system is beneficial for the manufacturer in ordinary and green markets and obtains the manufacturer’s optimal decisions. Basse Mama and Mandaroux [27] explored the extent to which cross-sectional differences in carbon dioxide emissions matter for future valuations of European firms regulated under the European Union Trading Scheme. Although the above literature has addressed governance on carbon emissions, to the best of our knowledge, they have not addressed the implications of carbon taxes that can be levied on different agents. As mentioned earlier, the outsourcing of remanufacturing usually involves different stakeholders. Thus, the outsourcing of remanufacturing is not related to different operations decision makers but creates different taxpayers for carbon taxes. To fill this gap, in this paper, we extend the above-mentioned literature to provide a detailed understanding of which taxpayers are beneficial for economic and/or environmental outcomes.

The second related stream is remanufacturing outsourcing. Wang et al. [28] investigated the optimal decisions for OEMs who outsource remanufacturing to the retailer and accept returns of remanufacturable products. Zhang et al. [14] developed two models in which OEMs produce new products but outsource remanufacturing operations to authorized remanufacturers, and they highlighted the potential strategies for dealing with the cannibalization from remanufacturing outsourcing. Zhao et al. [29] obtained a long-term outsourcing remanufacturing strategy under the information asymmetry caused by a third-party remanufacturer misreporting remanufacturing production costs. Zou et al. [30] found that when consumers perceive the remanufactured products to have a low value, the third-party remanufacturers prefer the authorization approach; otherwise, they prefer the outsourcing approach. We refer the interested reader to [31] for the related literature review. These authors have studied the economic and environmental issues under remanufacturing outsourcing, but they have not paid attention to how carbon taxes impact the relationships of remanufacturing outsourcing. However, more and more governments have realized that environmental, social, and governance performance has become financial in the long run. Therefore, to fill this gap, we intend to contribute to this stream by addressing the possible impacts of carbon taxes levied on different agents on remanufacturing outsourcing decisions.

Our paper is closely related to the literature on carbon taxes set to different agents. Chung et al. [21] presented a model of the strategic behavior of firms operating in a spatial supply chain network and found that in response to such changes in prices and exogenous environmental taxes, manufacturing firms may strategically alter a variety of choices, such as make–buy decisions, with respect to intermediate inputs. Joan et al. [22] specified what an optimal pollution tax should be when dealing with a vertical Cournot oligopoly and showed that an optimal environmental tax is always the result of a trade-off between two antagonistic effects. Our paper has two notable differences from these studies. First, as mentioned earlier, although they have addressed the implications of carbon taxes set to different agents, remanufacturing outsourcing has not been addressed. However, in the remanufacturing industry, many brand name OEMs, including Land Rover and IBM, have outsourced their remanufacturing operations to many selected IRs. Thus, we extended their models to the remanufacturing industry to provide a detailed account of how the carbon taxes levied on different agents impact the optimal decisions for remanufacturing outsourcing. Second, the relationships among different agents in our paper are quite different from those in the above literature. Previous authors have mainly focused on the relationships among different agents such as the manufacturer and its retailing firms. In contrast, we assumed that the manufacturer (referred to as the OEM in this paper) always outsources remanufacturing operations to IRs. Consequently, in addition to the cooperative relationship, OEMs contend with potential competition from IRs due to their products being confronted with cannibalization by remanufactured products. 

The final related stream of literature is in regard to sustainability issues in remanufacturing. Zheng et al. [32] adopted a multi-methodological approach and carried out a two-stage analysis of a three-player duopoly supply chain with a leader and two competitive followers who provide sustainable products or services. Meanwhile, Bai and Sarkis [33] introduced a new hybrid group decision method and integrated hesitant fuzzy set and regret theory for blockchain technology evaluation and selection. Similarly, Centobelli et al. [34] proposed the integrated Triple Retry framework for designing circular blockchain platforms. Sara et al. [35] explored how a variety of motivators and barriers are perceived by different companies from different industries. Although the above authors have studied sustainability issues under remanufacturing, they have not paid attention to how carbon taxes levied on different agents impact the relationships of remanufacturing outsourcing. As such, we intend to contribute to this stream by addressing the implications of a carbon tax mechanism in remanufacturing outsourcing related to sustainability issues.

## 3. Model Formulation and Solution

### 3.1. Problem Description

**Figure 1:**
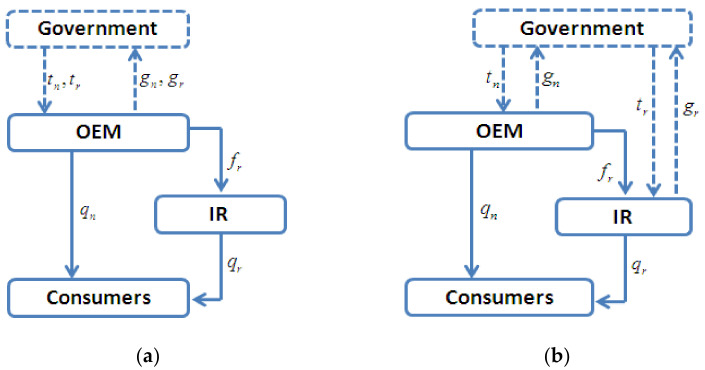
Problem structure: (**a**) Model C; (**b**) Model I.

We developed two game-theoretic models related to remanufacturing outsourcing with two possible options for carbon taxes: (1) acting as common brand owners, OEMs can be taxed for both new and remanufactured products (Model C, see Figure 1a; refers to the situation in which the carbon taxes related to remanufacturing are levied on the common brand owners of OEMs), or (2) acting as different polluting firms, the OEM is taxed only for new products; however, all carbon taxes related to remanufacturing are levied on the IRs (Model I, see Figure 1b; refers to the situation in which the carbon taxes related to remanufacturing are levied on IRs). 

These two theoretic models are consistent with the fact that the outsourcing of remanufacturing involves different stakeholders. For example, in the US remanufacturing industry, IRs account for 94% of the firms that engage in remanufacturing in the US market [16]. Many brand name OEMs, such as IBM, have created certification programs for their IRs, where all remanufactured products are inspected by their own engineers. As such, on the one hand, acting as a common brand owner, the OEM is still the ideal target for carbon emissions policies. Then, under this situation, the carbon taxes related to remanufacturing are levied on the common brand owners of OEMs. However, on the other hand, acting as the actual emitters for production and remanufacturing, as that in Model I, the OEMs and/or IRs should also be responsible for the carbon regulations of production and/or remanufacturing, respectively.

### 3.2. Methodologies and Assumptions

Given the framework in Section 3.1, we present the main symbols used in Table 2.

In the two game-theoretic models, the decision consequence is as follows. First, the OEM announces the patent license fees, $f_{r}$, and the carbon emission reduction levels (in Model C, the OEM chooses $g_{n}$ and $g_{r}$ for the new and remanufactured products, respectively; while in Model I, the OEM chooses $g_{n}$ for the new products, and the IR responds with $g_{r}$ for the remanufactured products). Following this, both the OEM and IR choose the optimal quantities of new products, $q_{n}$, and remanufactured products, $q_{r}$, simultaneously. To ensure the accuracy of the analysis, similar to [2,32,36], we adopt backward induction to identify the (subgame perfect) equilibria. 

All remanufactured products are made from used cores [14,29], and all products have two lives: one is a new product and the other is remanufactured. It should be noted that this assumption precludes remanufacturing cores that may be extracted from remanufactured products. This is consistent with the fact that all remanufactured cores obtained from remanufactured products would be built with outdated technology, and as such, few remanufacturers prefer to extract cores from remanufactured products [12,14,29].

The willingness to pay varies vertically across all consumers [36,37]. That is, keeping everything else constant, consumers prefer new products. This assumption reflects the fact that consumers are usually concerned about the quality of remanufactured products. For example, auction results from eBay show that the willingness to pay for a remanufactured consumer product is 15.3% lower than that for a new product [38]. We assume that the willingness to pay for a new product is uniformly distributed in the interval of [0, 1] [14,39,40]. To characterize the quality of remanufactured products as lower than that of new units, we assume that all consumers want discounted prices for remanufactured products compared to the prices for a new product, that is, δδu. Given the prices of new and remanufactured products $p_{n}$ and $p_{r}$, we determine that one consumer with a product type u can receive utilities from the new and remanufactured products that are $U_{n}=u-p_{n}$ and $U_{r}=\delta u-p_{r}$, respectively. Similar to Zhang et al. [14], Talat and Pietro [37], and Zhang and Zhang [36], we assumed that the market is constant over time and is normalized to 1. Then, consistent with the method of [14,36], we can obtain the demands for both products as follows.

(1) $$\begin{array}{l} p_{n}=1-q_{n}-\delta q_{r}\\ p_{r}=\delta (1-q_{n}-q_{r}) \end{array}$$

As in [12], in both models, the OEM is assumed to be a Stackelberg Leader who will produce all new products with a unit cost of $c_{n}$, but all remanufactured products are remanufactured by the IR with a unit cost of $c_{r}$. Similar to Yan et al. [12] and Wang et al. [23], to enable remanufacturing, a used product is less costly than producing a new one, and we assume that $c_{n}>c_{r}>0$. Let $t_{n}$($t_{r}$) denote the carbon tax rates of the new (remanufactured) products. Similar to Joan et al. [22] and Lin and Li [6], we take the carbon tax rates of $t_{n}$($t_{r}$) as exogenously determined, and let $t_{n}=t_{r}=t$. In addition, for the tax rate, t of the unit, the OEM has an incentive,$g_{n}$, to reduce carbon emissions. To characterize the diminishing returns on investment, similar to [41], we assume the cost structure gn=gr=Ik, where k is a scaling parameter that reflects the efficiency of the total investment of I. Similar forms of investment functions have been widely used in the literature on remanufacturing [2,14,41]. 

### 3.3. Model Solution

#### 3.3.1. Model C

As mentioned earlier, although the remanufacturing is outsourced to the IR, as with the new products, the OEM still controls the brands of the remanufactured products. Thus, acting as a common brand owner, in Model C, the OEM will be taxed for both new and remanufactured products. Thus, the OEM’s problems can be expressed as follows:

(2) $$\begin{array}{l} \underset{f_{r},g_{n},g_{r}}{\max}\pi_{m}^{C}=(p_{n}-c_{n})q_{n}+f_{r}q_{r}-\left(t-g_{n}\right)q_{n}-\left(t-g_{r}\right)q_{r}-kg_{n}^{2}-kg_{r}^{2}\\ \underset{q_{n}}{\max}\pi_{m}^{C}=(p_{n}-c_{n})q_{n}+f_{r}q_{r}-\left(t-g_{n}\right)q_{n}-\left(t-g_{r}\right)q_{r}-kg_{n}^{2}-kg_{r}^{2} \end{array}$$

Since the remanufacturing is undertaken by the IR, then the IR’s objective is to maximize its profits by choosing the units of remanufactured products, $q_{r}$. That is, given the optimal outcomes of $f_{r},g_{n},g_{r}$, the IR’s problem can be expressed as follows:

(3) $$\underset{q_{r}}{\max}\;\pi_{p}^{C}=\left(p_{r}-w_{r}-c_{r}\right)q_{r}$$

We use backward induction for the above problems. That is, we maximize the OEM’s and IR’s profits, yielding the optimal quantities of $q_{n}$, and $q_{r}$. Substituting the optimal quantities of both products into Equation and performing the function with the maximum yields $f_{r},g_{n},g_{r}$, we can obtain all optimal outcomes in Model C, which are summarized in Table 3 (all proofs are provided in the Appendix A).

#### 3.3.2. Model I

In Model I, acting as one of the polluting firms, the OEM is taxed only for new products; thus, we can obtain the OEM’s problem as follows:

(4) $$\begin{array}{l} \underset{f_{r},g_{n}}{\max}\;\pi_{m}^{I}=(p_{n}-c_{n})q_{n}+f_{r}q_{r}-\left(t-g_{n}\right)q_{n}-kg_{n}^{2}\\ \underset{q_{n}}{\max}\;\pi_{m}^{I}=(p_{n}-c_{n})q_{n}+f_{r}q_{r}-\left(t-g_{n}\right)q_{n}-kg_{n}^{2} \end{array}$$

On the other hand, because all carbon taxes related to remanufacturing are levied on the IRs, we can obtain the IR’s problem as follows:

(5) $$\underset{g_{r},q_{r}}{\max}\;\pi_{p}^{I}=\left(p_{r}-w_{r}-c_{r}\right)q_{r}-\left(t-g_{r}\right)q_{r}-kg_{r}^{2}$$

Using backward induction again, we can identify the optimal quantities $q_{n}^{I},q_{r}^{I}$ in Equation (4) and perform the function. With maximum yields $f_{r},g_{n},g_{r}$, we can obtain all optimal outcomes in Model I, which are summarized in Table 3.

## 4. Result and Discussion

### 4.1. Analysis of Optimal Decisions

We are in a position to address the first question of how carbon taxes levied on different agents impact the optimal decisions for remanufacturing outsourcing. In this section, we compare the optimal quantities of both products (the proof provided in the Appendix B).

**Proposition 1**. *The optimal quantities of remanufactured (new) products in Model C are always lower (higher) than that of Model I, that is, *$q_{r}^{C*}<q_{r}^{I*}(q_{n}^{C*}>q_{n}^{I*})$.

Proposition 1 suggests that when the carbon tax of remanufacturing is paid by the IR, the IR will offer more units of remanufactured products than in Model C. Note that in both models, remanufacturing is the only source for the IR. Furthermore, in Model I, the carbon tax of remanufacturing is paid by the IR, and the carbon tax on new products is undertaken by the OEM. However, in Model C, the OEM is the only taxpayer, and it is responsible for the carbon taxes of both products. Therefore, when the IR pays for the carbon tax of remanufacturing in Model I, the remanufacturing cost increases. Since the IR is the only source for remanufacturing, to maximize the profitability, the IR will offer more units of remanufactured products to deal with the increase in the remanufacturing costs. That is, the optimal quantities of remanufactured products in Model C are always lower than that of Model I, i.e., $q_{r}^{C*}<q_{r}^{I*}$.

On the other hand, confronted by the increased units of remanufactured products offered by the IR, the potential for the cannibalization of new product sales by remanufactured products increases. This may lead to the potential market and the marginal profits both being lower than those in Model C. Therefore, in Model I, confronted by the fiercer competition of the remanufactured products, the OEM has no choice but to provide fewer units of new products. That is, the optimal quantities of new products in Model C are always higher than that of Model I, i.e., $q_{n}^{C*}>q_{n}^{I*}$.

**Figure 2:**
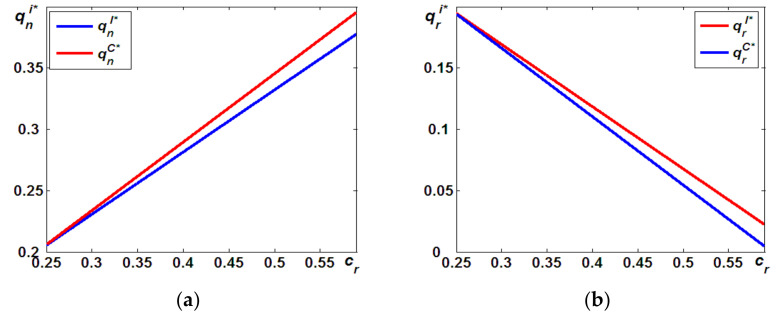
Differences in optimal quantities: (a) $q_{n}^{I*}$ vs. $q_{n}^{C*}$; (**b**) $q_{r}^{I*}$ vs. $q_{r}^{C*}$.

To better understand Proposition 1, we went a step further and used numerical experiments to simulate how remanufacturing costs impact the differences in the optimal quantities of both products. According to the methods by [6,15,22,23], in all numerical experiments, $c_{n}=0.8$, δ=0.9, t=0.3, and k=0.2. Thus, we found that, as shown in Proposition 1, the optimal quantities of new products in Model C are always higher than those in Model I, that is, $q_{n}^{C*}>q_{n}^{I*}$ (see Figure 2a). Meanwhile, Figure 2b indicates that the optimal quantities of remanufactured products in Model C are always lower than those in Model I, that is, $q_{r}^{C*}<q_{r}^{I*}$. Additionally, we could further conclude that, on the one hand, as indicated in Figure 2b, $q_{r}^{C*}$ and $q_{r}^{I*}$ both decrease the remanufacturing cost of $c_{r}$. Furthermore, the rate of decrease in $q_{r}^{C*}$ is greater than that of $q_{r}^{I*}$, and as a result, the difference in the optimal quantities of remanufactured products increases the remanufacturing cost of $c_{r}$. On the other hand, as shown in Figure 2a, $q_{n}^{C*}$ and $q_{n}^{I*}$ both increase in the remanufacturing cost of $c_{r}$. Furthermore, the rate of increase in $q_{n}^{C*}$ is greater than that of $q_{n}^{I*}$, and as a result, the difference in the optimal quantities of new products increases the remanufacturing cost of $c_{r}$.

**Figure 3:**
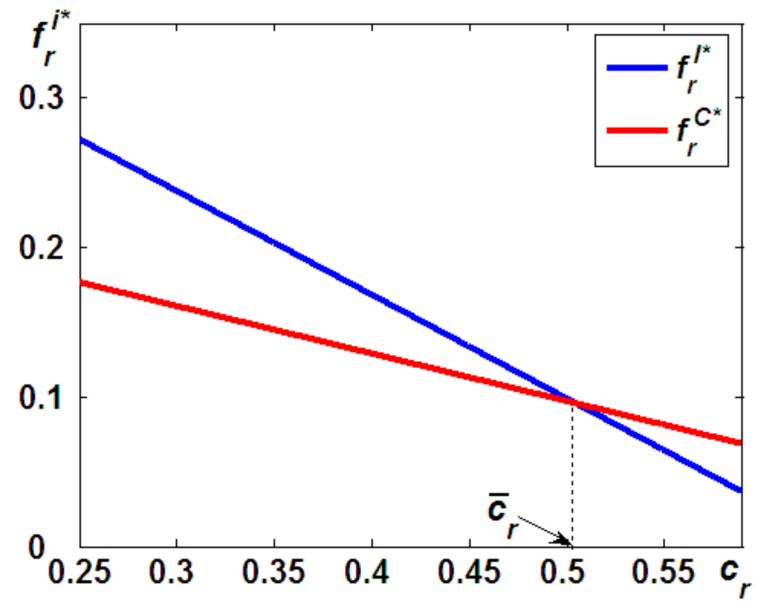
Differences in the relicensing fees.

Based on the analysis in Proposition 1, one may expect the OEM to increase the relicensing fees that are charged in Model I to limit the increased cannibalization problems from IR’s remanufacturing. In fact, this expectation is not always true, as illustrated in Figure 3, so we offer the following proposition (the proof provided in the Appendix C).

**Proposition 2**. *The OEM will charge a higher relicensing fee for the IR’s remanufacturing in Model I, i.e.,* $f_{r}^{C}<f_{r}^{I}$ *, if* $c_{r}<{\bar{c}}_{r}$ *; otherwise, the opposite is true.*

In both models, there are two main profit sources for the OEM: one is selling new products and the other are the relicensing fees charged for remanufacturing outsourcing. Recall that, in Model I, the carbon taxes of remanufactured products are paid by the IR, and the carbon taxes of new products are undertaken by the OEM. Thus, if the remanufacturing cost is relatively low, i.e., $c_{r}<{\bar{c}}_{r}$, remanufacturing is a profitable business. Observing the profitable business of remanufacturing, to maximize its own profits, the OEM will transfer some profits from the remanufacturing at the IR by charging a higher relicensing fee. 

On the other hand, when the remanufacturing cost is relatively high, i.e., $c_{r}>{\bar{c}}_{r}$*,* remanufacturing operations are less profitable than selling new products. Hence, to maximize the profitability, the OEM will charge a lower relicensing fee for the IR’s remanufacturing in Model I, i.e., $f_{r}^{I}<f_{r}^{C}$, to support the IR’s remanufacturing operations when the IR is responsible for the carbon taxes in Model I.

Proposition 1 indicates that, when compared to Model C, in Model I, the OEM is confronted with fiercer competition from the remanufactured products, which leads to fewer units of new products available in the market, resulting in potentially lower profits from new product sales. However, it should be noted that when the remanufacturing operations are outsourced to an IR, the remanufacturing is also a possible source for the OEM. Proposition 2 states that when the remanufacturing operation is outsourced to an IR, the OEM can transfer some profits from the remanufacturing at the IR by charging a different relicensing fee. This is consistent with the fact that IBM created certification programs for its IRs, where all remanufactured products are inspected by IBM engineers. As a result, IBM has not only extended producer responsibility for all remanufactured equipment but has also obtained a substantial relicensing fee for remanufacturing outsourcing. 

### 4.2. Analysis of Economic Performance

**Figure 4:**
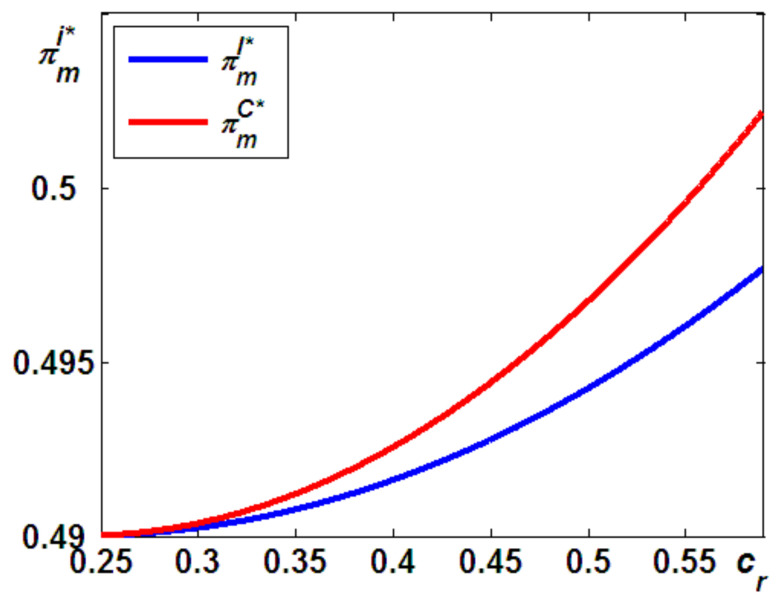
Difference in the OEM’s profits.

Here, we address the second question that was posed at the beginning of this paper: which taxpayer is more beneficial for economic and/or environmental outcomes? In this subsection, as shown in Figure 4, we first answer the question of which taxpayer is better for the economic outcomes, as follows (the proof provided in the Appendix D).

**Proposition 3**. *The OEM’s profits in Model C are always higher than those in Model I, that is,*$\pi_{m}^{C*}>\pi_{m}^{I*}$.

Proposition 3 states that the OEM will always benefit more from Model C. This can be interpreted as follows: as shown in Proposition 1, in Model I, when the carbon tax of remanufacturing is paid by the IR, the IR will offer more units of the remanufactured products than in Model C. It should be noted that the more units of remanufactured products there are, the more cannibalization problems arise. This will lead to a potentially lower market and lower marginal profits for the new products than in Model C. Thus, although the OEM has to pay the carbon emission taxes in Model C, its profits in Model I are always lower than those in Model C, due to the higher cannibalization of new products sales by remanufactured products.

Numerous studies have found that the cannibalization problems from remanufactured products hurt the profitability of new products. For example, Ferguson and Toktay [42] suggested that although remanufacturing end-of-life products may backfire for manufacturers operating in industries, such businesses are attractive to third-party remanufacturers who may seriously cannibalize the sales of the original manufacturer. In addition, Chai et al. [19] found that the remanufacturing outsourced to IRs decreased the production quantity of new products and the total market volume. In this study, we extend the above-mentioned literature to provide a detailed understanding of which taxpayer is more beneficial for economic profitability. Unlike the above literature, in both of our models, the remanufacturing is always outsourced to the IR. Given this channel structure, we went a step further and found that the OEM will always benefit more from the IR undertaking the carbon tax.

We now answer the question of which taxpayer is better for the economic performance of the IR (the proof provided in the Appendix E).

**Proposition 4**. *The IR’s profits in Model C are always lower than those in Model I, that is,*$\pi_{p}^{C*}<\pi_{p}^{I*}$.

Proposition 4 suggests that the IR’s profitability will be hurt if the OEM acted as the taxpayer of the carbon emission tax. Note that in both models, the OEM is assumed to be a Stackelberg Leader who charges a unit patent licensing fee for remanufacturing outsourcing. In Model C, when the OEM acts as a taxpayer for the carbon emission tax, as Proposition 1 shows, to cover the additional carbon emission taxes of remanufacturing, the OEM will provide higher quantities of new products. This will limit the quantities of remanufactured products and naturally hurt the profitability of remanufacturing. As such, the IR’s profitability in Model C is always lower than that in Model I, that is, $\pi_{p}^{C*}<\pi_{p}^{I*}$.

**Figure 5:**
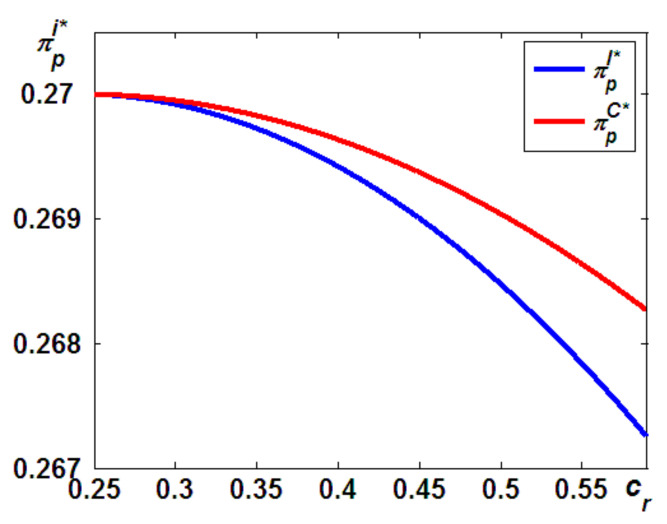
Differences in the IR’s profits.

Figure 5 shows a detailed illustration of Proposition 4. On the one hand, as the remanufacturing cost increases, the IR’s quantities in Model C and Model I both decrease. Lower quantities of remanufactured products are harmful to the profitability of remanufacturing, whether the carbon emission taxes are levied on the OEM or the IRs. On the other hand, as the remanufacturing cost increases, the rate of decrease in Model C is always greater than that in Model I. This can be interpreted as follows: confronted with the higher quantities of units of new products offered by the OEM, the potential marginal profits in Model C are lower than that in Model I. Thus, as Figure 5 illustrates, as the remanufacturing cost increases, the rate of decrease in the marginal profits in Model C is always greater than that in Model I. 

Based on Propositions 1, 2, 3, and 4, we can conclude that for firms related to remanufacturing outsourcing, they should undertake their own carbon emission taxes because this allows the taxpayer to choose to produce more units of its preferred products and leaves its rival at a huge disadvantage. More specifically, as Proposition 1 shows, when the OEM undertakes the carbon emission tax, it will provide more units of new products, which results in the lower profitability of the IR (see Proposition 3). Meanwhile, when the IR acts as the taxpayer, as indicated in Proposition 2, it will offer more units of remanufactured products, which leads to the lower profitability of the OEM (see Proposition 4).

### 4.3. Analysis of Environmental Sustainability

In this subsection, we highlight the difference in environmental sustainability between the models. More specifically, we determine which taxpayer is better for environmental performance. 

In both models, for any tax rate, t, per unit, the incentives of $g_{n}$ and $g_{r}$ are assumed to reduce the carbon emissions for new and remanufactured products, respectively. We first compare the difference in the above incentives for both models as follows (the proof provided in the Appendix F).

**Proposition 5**. *In Model C, the incentive for reducing the carbon emissions per unit of new (remanufactured) products is always higher (lower) than that in Model I, that is,* $g_{n}^{C*}>g_{n}^{I*}$ *(* $g_{r}^{C*}<g_{r}^{I*}$ *).*

**Figure 6:**
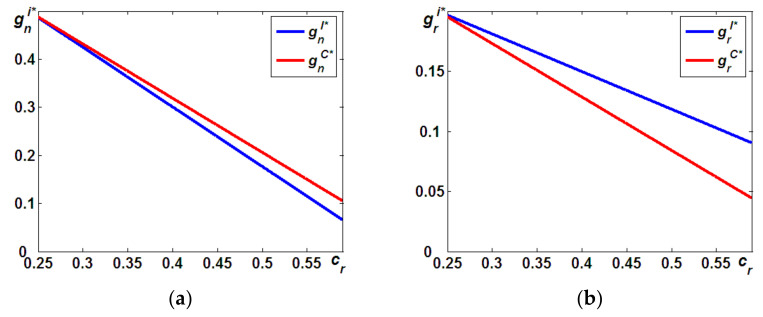
Differences in incentives for carbon emission reduction. (a) $g_{n}^{I*}$ vs. $g_{n}^{C*}$; (b) $g_{r}^{I*}$ vs. $g_{r}^{C*}$.

Proposition 5 indicates that when the OEM acts as a taxpayer for the carbon emissions of both products, the OEM will more likely have a higher incentive to reduce the carbon emissions per new product, while it has less incentive to reduce the carbon emissions per remanufactured product. Note that the total reduction of carbon emissions equals the units of new and remanufactured products multiplied by the incentive of the reduced emissions per unit. Proposition 1 states that in Model C, when the OEM acts as the taxpayer for the carbon emissions of both products, the OEM will provide higher quantities of new products but fewer units of remanufactured products. As such, confronted with the additional cost induced by the carbon emissions, the OEM will have a higher incentive to reduce the carbon emissions per new product because it can reduce the total carbon emissions efficiently (see Figure 6a). On the other hand, as Proposition 2 shows, the OEM will limit the availability of remanufactured products to reduce the cannibalization problem from remanufacturing. Thus, confronted with fewer quantities of remanufactured products, the IR has a lower incentive to reduce the carbon emissions per remanufactured product because it cannot reduce the total carbon emissions efficiently (see Figure 6b).

Based on Propositions 1 and 5, we now compare the differences in total carbon emission reductions of both models and address the last question regarding which taxpayer is better for environmental performance. This is summarized in the following proposition (the proof provided in the Appendix G).

**Proposition 6**. *The levels of carbon emission reduction in Model C are lower than those in Model I, if* $c_{r}>\Delta c_{r}$ *; otherwise, the opposite is true.*

**Figure 7:**
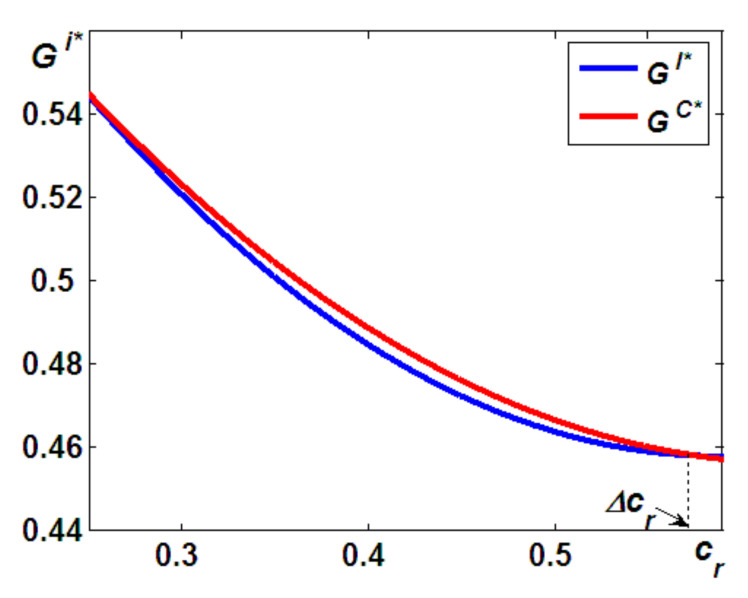
Differences in the total carbon emission reduction.

Proposition 6 can be interpreted as follows. As Figure 1b indicates, the higher the costs are for remanufacturing, the fewer units of remanufactured products will be available in the market. In addition, when the costs are high, i.e., $c_{r}>\Delta c_{r}$, the potential for cannibalization of new product sales by remanufactured products decreases. Thus, confronted by a weaker cannibalization problem due to remanufacturing, as shown in Proposition 1, the OEM will provide more units of new products. On the other hand, as indicated in Proposition 5, in Model c, the incentive to reduce the carbon emissions of new (remanufactured) products is always higher (lower) than that in Model I. Thus, as Figure 7 illustrates, when $c_{r}>\Delta c_{r}$, the levels of carbon emission reduction in Model C are lower than those in Model I because the total reduction in carbon emissions to equal the units of new products and remanufactured products is multiplied by the incentives of the reduced emissions per unit.

Based on Propositions 1, 2, 5, and 6, we can conclude that on the one hand, carbon emission taxes can incentivize the emitter to reduce the carbon emissions per unit (see Proposition 5); however, environmental groups and agencies should also give their attention to volume effects (see Propositions 1 and 2). On the other hand, for environmental groups and agencies, if they set carbon taxes to different agents, as shown in Proposition 6, they should charge the carbon taxes to the OEM when the costs for remanufacturing are $c_{r}>\Delta c_{r}$; otherwise, they should pay attention to the IRs. 

## 5. Conclusions, Discussion, and Future Research Opportunities

The traditional industrial structure has witnessed an increase in greenhouse gas emissions that endangers our daily lives. Therefore, we need to impose some additional policies to reform the traditional industrial structure to offset its negative effects. In practice, to reduce carbon dioxide emissions, some governments have imposed technology standards, emission trading systems, deposit systems, carbon taxes, etc. Among these methods, a carbon tax is highly recommended because it is a cost-effective instrument for achieving a given abatement target [5,6]. As we know, the taxes levied on different agents would induce strategic outcomes for optimal decisions [21,22].

In recent decades, due to the consumption of fewer natural resources and energy than production, more and more OEMs have developed remanufacturing as an integral part of their existing business systems. In practice, to relieve the pressure from carbon taxes and improve social welfare, some OEMs lacking remanufacturing expertise have outsourced their remanufacturing operations to independent remanufacturers According to a survey of the US remanufacturing industry, IRs account for 94% of firms that engage in remanufacturing in the US market [16]. Obviously, the above remanufacturing outsourcing usually involves different stakeholders. 

This raises the important question of what are the implications of carbon taxes levied on remanufacturing outsourcing decisions? As such, we developed two models related to remanufacturing outsourcing with two possible options for carbon taxes: (1) acting as common brand owners, OEMs can be taxed for both new and remanufactured products (Model C), or (2) acting as different polluting firms, the OEM is taxed only for new products; however, all carbon taxes related to remanufacturing are levied on IRs (Model I). Using these two models, we highlight the following implications of carbon taxes levied on remanufacturing outsourcing decisions: How do carbon taxes levied on different agents impact the optimal decisions for remanufacturing outsourcing? Which taxpayer is more beneficial for economic and/or environmental outcomes?

### 5.1. Discussion of Theoretical Implications

From the theoretical perspective, this paper makes two main contributions. First, although numerous studies have highlighted the roles of different agents regarding remanufacturing outsourcing (see, e.g., Zhang et al. [13], Chai et al. [18], and Huang et al. [19]), little attention has been paid to the implications of carbon taxes levied on different agents on remanufacturing outsourcing decisions. Conversely, several studies (see, e.g., Chung et al. [20] and Joan et al. [21]) have recently highlighted the fact that many governments have adopted carbon taxes set to different agents; however, remanufacturing outsourcing is not addressed. As such, our model fills this gap by highlighting the implications of carbon taxes on remanufacturing outsourcing decisions. Second, our analysis provides a detailed understanding of sustainable strategic management in remanufacturing outsourcing under a carbon tax mechanism. We found that a carbon emission tax indeed leads to the emitter reducing their carbon emissions per unit; however, the levels of carbon emission reduction are dependent on the remanufacturing costs. More specifically, the levels of carbon emissions reduction in Model C are lower than those in Model I, if $c_{r}>\Delta c_{r}$; otherwise, the opposite is true. In addition, our results also provide clarity on the implications related to optimal decisions. Specifically, when the OEM undertakes the carbon emission tax, it will provide more units of new products (see Proposition 1). Meanwhile, when the IR acts as the taxpayer, as indicated in Proposition 2, it will offer more units of remanufactured products.

### 5.2. Discussion of Managerial Implications

Our analysis provides several important managerial insights for managers and environmental agencies. Our results revealed that when the OEM undertakes the carbon emission tax, it leads to the OEM providing more units of new products. More units of new products will limit the units of remanufactured products and result in lower profitability of the IR. However, when the IR acts as the taxpayer, it will offer more units of remanufactured products. The more units there are of remanufactured products in Model I will lead to lower profitability for the OEM. As such, from the economic performance perspective, we suggest that firms undertake the carbon emission taxes themselves because this allows the taxpayer to choose more units for its preferred products and leaves its rival at a huge disadvantage. Moreover, from the environmental sustainability angle, carbon emission taxes indeed lead to mitigating effects on the carbon emissions per unit; however, carbon emission taxes can result in firms having a higher incentive to offer more units to maximize their own profit. As such, our analysis suggests that environmental groups and agencies should not only highlight the reduction of carbon emissions per unit but should also pay attention to reducing the volume effect.

### 5.3. Future Research Opportunities

The research in this paper can be extended in the following directions. First, we developed a two-stage model with one OEM and one IR; however, in practice, OEMs may outsource remanufacturing to multiple IRs. Therefore, future research can go a step further to highlight the possible competition in the remanufacturing industry. Second, in both models, the OEM is assumed to be a Stackelberg Leader who can make efforts to control cannibalization problems between both products, whereas in recent years, many new technologies, such as blockchain, may help improve this goal by managing inventory, inventory, material flows, warehousing, designing products and services, delivery, and payment [33,35]. Thus, it would be interesting to explore how the application of our main results converges or diverges from other approaches. Third, in both models, we assume the carbon emission tax is exogenous; however, in practice, the regulatory agency has the power to set the carbon emission tax or pollution fine levels. Finally, the assumptions, including complete information, a single period model, and a linear relicensing fee, can be relaxed in future research.

## Figures and Tables

**Table 1:** A quick overview of our contributions to the literature.

	Governance on Carbon Emissions	Remanufacturing Outsourcing	Carbon Taxes	Sustainability Issues
Wang, et al. [23], Zhou, et al. [24], Liu, et al. [25], Chai, et al. [26], Basse Mama and Mandaroux [27]	√	×	×	×
Wang, et al. [28], Zhao, et al. [29], Zhang, et al. [14], Zou, et al. [30]	×	√	×	×
Chung, et al. [21], Joan, et al. [22]	×	×	√	×
Zheng, et al. [32], Bai and Sarkis [33], Centobelli, et al. [34], Sara, et al. [35]	×	×	×	√
This paper	√	√	√	√

Note: √ means yes, × means no.

**Table 2:** Main symbols in this paper.

Symbol	Definition
$c_{n}/c_{r}$	Unit cost for producing/remanufacturing
δ	Value of discount for remanufactured products
$q_{n}/q_{r}$	Quantities of new/remanufactured products
t	Tax rate of carbon emissions
$f_{r}$	Unit patent license fee for remanufacturing outsourcing
$g_{n}/g_{r}$	Levels of incentive for carbon emission reductions of new/remanufactured products
$\pi_{j}^{i}$	Player j’s profit in Model i, where j∈(m,p) denotes the OEM and I, while i∈(C,I) refers to Models C and I
k	Scaling parameter of investment

**Table 3:** Equilibrium decisions and profits.

Optimal Outcomes of Model C
$\begin{array}{l} g_{n}^{C}=\frac{1+k\delta^{2}c_{n}+k\delta^{2}t+8k\delta c_{n}+4k\delta t+3k\delta^{2}-c_{n}-t-8k\delta -4k\delta c_{r}}{12k^{2}\delta^{2}+k\delta^{2}+8k\delta +4k-32k^{2}\delta -1}\\ g_{r}^{C}=\frac{\delta -4k\delta c_{n}-4k\delta t-c_{r}+4kc_{r}+4kt-t}{12k^{2}\delta^{2}+k\delta^{2}+8k\delta +4k-32k^{2}\delta -1} \end{array}$ $f_{r}^{C}=\frac{\left[\begin{array}{l} c_{r}-\delta +2k\delta +4k\delta^{2}-4kc_{r}-4k\delta c_{r}+2k\delta c_{n}+6k\delta t-16k^{2}\delta^{2}+6k^{2}\delta^{3}\\ +k\delta^{2}t+2k^{2}\delta^{3}c_{n}+2k^{2}\delta^{3}t-8k^{2}\delta^{2}c_{r}+4k^{2}\delta^{2}t-16tk^{2}\delta +16c_{r}k^{2}\delta \end{array}\right]}{12k^{2}\delta^{2}+k\delta^{2}+8k\delta +4k-32k^{2}\delta -1}$ $q_{n}^{C}=\frac{(6k\delta^{2}-2k\delta^{2}t-2k\delta^{2}c_{n}+\delta^{2}-\delta t-16k\delta -4k\delta c_{r}+12k\delta t-\delta c_{r}+16k\delta c_{n}+2-2c_{n}-2t)k}{12k^{2}\delta^{2}+k\delta^{2}+8k\delta +4k-32k^{2}\delta -1}$ $q_{r}^{C}=\frac{-2\left(4k\delta c_{n}+4k\delta t-\delta +t-4kc_{r}-4tk+c_{r}\right)k}{12k^{2}\delta^{2}+k\delta^{2}+8k\delta +4k-32k^{2}\delta -1}$ $\pi_{m}^{C}=(1-q_{n}^{C*}-\delta q_{r}^{C*}-c_{n})q_{n}^{C*}+f_{r}^{C*}q_{r}^{C*}-\left(t-g_{n}^{C*}\right)q_{n}^{C*}-\left(t-g_{r}^{C*}\right)q_{r}^{C*}-kg_{n}^{C^{*2}}-kg_{r}^{C^{*2}}$ $\pi_{p}^{C}=\frac{4k^{2}\delta {\left(4k\delta t-\delta +4k\delta c_{n}+t+c_{r}-4tk-4kc_{r}\right)}^{2}}{{\left(12k^{2}\delta^{2}+k\delta^{2}+8k\delta +4k-32k^{2}\delta -1\right)}^{2}}$
**Optimal Outcomes of Model I**
$\begin{array}{l} g_{n}^{I}=\frac{\left[\begin{array}{l} 4c_{n}-4+4t-4k\delta^{2}c_{r}-32k\delta c_{n}+3k\delta^{3}-16k\delta t+\\ 4k\delta^{2}c_{n}+32k\delta -20k\delta^{2}+k\delta^{3}t+k\delta^{3}c_{n}+16k\delta c_{r} \end{array}\right]}{128k^{2}\delta -16k-80k^{2}\delta^{2}+4+4k\delta^{2}+12k^{2}\delta^{3}+k\delta^{3}-32k\delta }\\ g_{r}^{I}=\frac{4(c_{r}+t-\delta +4k\delta c_{n}+4k\delta t-4kc_{r}-4kt)}{128k^{2}\delta -16k-80k^{2}\delta^{2}+4+4k\delta^{2}+12k^{2}\delta^{3}+k\delta^{3}-32k\delta } \end{array}$ $f_{r}^{I}=\frac{\left[\begin{array}{l} 2k\delta (2\delta^{2}-4-8\delta -2c_{r}\delta -2\delta t+4c_{n}+8c_{r}+12t\\ +3k\delta^{3}-20k\delta^{2}+32k\delta -4k\delta^{2}c_{r}+24k\delta t-4k\delta^{2}c_{n}\\ +k\delta^{3}t+k\delta^{3}c_{n}+24k\delta c_{r}-32kt-8k\delta^{2}t-32kc_{r}) \end{array}\right]}{128k^{2}\delta -16k-80k^{2}\delta^{2}+4+4k\delta^{2}+12k^{2}\delta^{3}+k\delta^{3}-32k\delta }$ $q_{n}^{I}=\frac{\left[\begin{array}{l} -(2k\delta^{3}c_{n}-\delta^{3}-6k\delta^{3}+2k\delta^{3}t-24k\delta^{2}c_{n}-20k\delta^{2}t+\delta^{2}t+4\delta^{2}+40k\delta^{2}+\delta^{2}c_{r}\\ +4k\delta^{2}c_{r}-16k\delta c_{r}-4c_{r}\delta -64k\delta +48k\delta t+64k\delta c_{n}-4\delta t-8c_{n}-8t+8)k \end{array}\right]}{128k^{2}\delta -16k-80k^{2}\delta^{2}+4+4k\delta^{2}+12k^{2}\delta^{3}+k\delta^{3}-32k\delta }$ $q_{r}^{I}=\frac{2\left(4-\delta \right)\left(c_{r}+t-\delta +4k\delta c_{n}+4k\delta t-4kc_{r}-4tk\right)k}{128k^{2}\delta -16k-80k^{2}\delta^{2}+4+4k\delta^{2}+12k^{2}\delta^{3}+k\delta^{3}-32k\delta }$ $q_{r}^{I}=\frac{2\left(4-\delta \right)\left(c_{r}+t-\delta +4k\delta c_{n}+4k\delta t-4kc_{r}-4tk\right)k}{128k^{2}\delta -16k-80k^{2}\delta^{2}+4+4k\delta^{2}+12k^{2}\delta^{3}+k\delta^{3}-32k\delta }$ $\pi_{m}^{I}=(1-q_{n}^{I*}-\delta q_{r}^{I*}-c_{n})q_{n}^{I*}+f_{r}^{I*}q_{r}^{I*}-\left(t-g_{n}^{I*}\right)q_{n}^{I*}-kg_{n}^{I^{*2}}$ $\pi_{p}^{I}=\frac{4k(16k\delta -4+k\delta^{3}-8k\delta^{2}){\left(c_{r}+t-\delta +4k\delta c_{n}+4k\delta t-4kc_{r}-4tk\right)}^{2}}{128k^{2}\delta -16k-80k^{2}\delta^{2}+4+4k\delta^{2}+12k^{2}\delta^{3}+k\delta^{3}-32k\delta }$

## Data Availability

Not applicable.

## Appendix A. Derivation of the Optimal Decisions

### Appendix A.1. Derivation of the Optimal Decisions under Model C

After plugging Equation (1) into the Equations (2) and (3) and solving the first-order condition with $q_{n}$ and $q_{r}$, we can then obtain $q_{n}^{C}=\frac{\delta -2-w_{r}-c_{r}+2c_{n}+2t-2g_{n}}{\delta -4}$ and $q_{r}^{C}\;=\frac{\delta -2w_{r}-2c_{r}+\delta c_{n}+\delta t-\delta g_{n}}{\delta \left(4-\delta \right)}$

After plugging $q_{n}^{C*}$ and $q_{r}^{C*}$,we can rewrite the manufacturer’s profit (2) and

$$\max_{f_{r},g_{n},g_{r}}\;\pi_{m}^{C}=\frac{\left[\begin{array}{cc} \; & 4g_{n}\delta^{2}-8w_{r}\delta -4c_{r}\delta -4\delta^{2}c_{n}-5\delta^{2}t+3w_{r}\delta^{2}-3w_{r}^{2}\delta +8w_{r}^{2}-\delta^{2}t^{2}\\ \; & -4\delta c_{n}^{2}-8kg_{r}^{2}\delta^{2}-\delta c_{r}^{2}+kg_{r}^{2}\delta^{3}-\delta^{3}-8kg_{n}^{2}\delta^{2}+kg_{n}^{2}\delta^{3}-2g_{r}c_{r}\delta \\ \; & -2g_{r}w_{r}\delta +2c_{r}\delta^{2}-g_{r}g_{n}\delta^{2}+g_{r}\delta^{2}t-4g_{n}^{2}\delta +g_{r}\delta^{2}c_{n}+4g_{r}g_{n}\delta \\ \; & -4g_{r}\delta c_{n}+4\delta c_{r}c_{n}-\delta^{2}tc_{n}+\delta^{2}tg_{n}+8g_{n}\delta c_{n}-4g_{n}\delta c_{r}+4\delta tg_{n}-4\delta tc_{n}\\ \; & +6\delta tc_{r}-w_{r}g_{n}\delta^{2}+2w_{r}\delta t+4\delta^{2}+w_{r}\delta^{2}t+w_{r}\delta^{2}c_{n}-4w_{r}c_{r}\delta +g_{r}\delta^{2}\\ \; & +8g_{r}w_{r}+8g_{r}c_{r}-4g_{r}\delta -4\delta -8w_{r}\delta -8c_{r}t+8\delta c_{n}+12\delta t-8g_{n}\delta \\ \; & +8w_{r}c_{r}+16kg_{r}^{2}\delta +16kg_{n}^{2}\delta -4g_{r}\delta t \end{array}\right]}{-\delta {\left(\delta -4\right)}^{2}}$$

For the above function, the first derivatives of profit are

$$\frac{\partial \pi_{m}^{C}}{\partial f_{r}}=\frac{\left[\begin{array}{l} 2\delta t-8t-8\delta +8g_{r}+\delta^{2}c_{n}+\delta^{2}t-4c_{r}\delta \\ 8c_{r}-2g_{r}\delta -g_{n}\delta^{2}-6w_{r}\delta +3\delta^{2}+16w_{r} \end{array}\right]}{-\delta {\left(\delta -4\right)}^{2}}$$

$$\frac{\partial \pi_{m}^{C}}{\partial g_{r}}=\frac{2w_{r}-\delta +2c_{r}-\delta c_{n}-\delta t+g_{n}\delta }{\delta {\left(\delta -4\right)}^{2}}-2kg_{r}$$

$$\frac{\partial \pi_{m}^{C}}{\partial g_{n}}=\frac{\left[\begin{array}{l} 8-8c_{n}+4c_{r}-4t-\delta t+8g_{n}-4\delta -4g_{r}\\ +g_{r}\delta +w_{r}\delta -2kg_{n}\delta^{2}+16kg_{n}\delta -32kg_{n} \end{array}\right]}{{\left(\delta -4\right)}^{2}}$$

and the Hessian is

$$H=\left(\begin{array}{ccc} \frac{2(3\delta -8)}{\delta {\left(\delta -4\right)}^{2}} & \frac{2}{\delta (\delta -4)} & \frac{\delta }{{\left(\delta -4\right)}^{2}}\\ \frac{2}{\delta (\delta -4)} & -2k & \frac{1}{\delta -4}\\ \frac{\delta }{{\left(\delta -4\right)}^{2}} & \frac{1}{\delta -4} & \frac{8-2k\delta^{2}+16k\delta -32k}{{\left(\delta -4\right)}^{2}} \end{array}\right)$$

The conditions for profit maximization will be satisfied if

$$k>\frac{-4-8\delta -\delta^{2}-\sqrt{16-64\delta +120\delta^{2}+16\delta^{3}+\delta^{4}}}{2(12\delta^{2}-32\delta )}$$

The equilibrium decisions are obtained by solving the system of three equations of the OEM yields:

$$\begin{array}{l} g_{n}^{C}=\frac{1+k\delta^{2}c_{n}+k\delta^{2}t+8k\delta c_{n}+4k\delta t+3k\delta^{2}-c_{n}-t-8k\delta -4k\delta c_{r}}{12k^{2}\delta^{2}+k\delta^{2}+8k\delta +4k-32k^{2}\delta -1}\\ g_{r}^{C}=\frac{\delta -4k\delta c_{n}-4k\delta t-c_{r}+4kc_{r}+4kt-t}{12k^{2}\delta^{2}+k\delta^{2}+8k\delta +4k-32k^{2}\delta -1} \end{array}$$

$$f_{r}^{C}=\frac{\left[\begin{array}{l} c_{r}-\delta +2k\delta +4k\delta^{2}-4kc_{r}-4k\delta c_{r}+2k\delta c_{n}+6k\delta t-16k^{2}\delta^{2}+6k^{2}\delta^{3}\\ +k\delta^{2}t+2k^{2}\delta^{3}c_{n}+2k^{2}\delta^{3}t-8k^{2}\delta^{2}c_{r}+4k^{2}\delta^{2}t-16tk^{2}\delta +16c_{r}k^{2}\delta \end{array}\right]}{12k^{2}\delta^{2}+k\delta^{2}+8k\delta +4k-32k^{2}\delta -1}$$

Substituting the optimal outcomes of $f_{r}^{C},g_{n}^{C},g_{r}^{C}$ into $q_{n}^{C},q_{r}^{C}$, we can obtain all optimal outcomes of Model C in Table 3.

### Appendix A.2. Derivation of the Optimal Decisions under Model I

Plugging (1) into the Equations (4) and (5) into the OEM’s and IR’s profit and maximizing them with $q_{n}^{I}$, and $q_{r}^{I}$ yields

$$\begin{array}{l} q_{n}^{I}=\frac{\delta -2+t-w_{r}-c_{r}+g_{r}+2c_{n}-2g_{n}}{\delta -4}\\ q_{r}^{I}=\frac{\delta -2t-2w_{r}-2c_{r}+\delta t+2g_{r}+\delta c_{n}-g_{n}\delta }{\delta (4-\delta )} \end{array}$$

After plugging both optimal quantities into Equations (4) and (5), we can rewrite the OEM’s and IR’s profit as follows:

$$\max_{f_{r},g_{n}}\;\pi_{m}^{I}=\frac{\left[\begin{array}{cc} \; & 4\delta tc_{n}-w_{r}\delta^{2}t-4w_{r}g_{r}\delta -4\delta^{2}+4c_{r}\delta +2\delta^{2}t-4g_{r}\delta +4\delta^{2}c_{n}\\ \; & -4g_{n}\delta^{2}-2\delta c_{r}g_{r}+2\delta tg_{r}-4\delta tg_{n}+4\delta g_{r}c_{n}+4\delta +4g_{n}\delta c_{r}\\ \; & -4g_{n}\delta g_{r}-8g_{n}\delta c_{n}-8w_{r}^{2}-2\delta tc_{r}-4\delta c_{r}c_{n}-w_{r}\delta^{2}c_{n}+8w_{r}\delta \\ \; & +w_{r}g_{n}\delta^{2}-4\delta t-8\delta c_{n}+8g_{n}\delta +\delta^{3}+\delta t^{2}+4g_{n}^{2}\delta -2c_{r}\delta^{2}\\ \; & +2g_{r}\delta^{2}+\delta c_{r}^{2}+\delta g_{r}^{2}+4\delta c_{n}^{2}-3w_{r}\delta^{2}+3w_{r}^{2}\delta -8w_{r}t-8w_{r}c_{r}\\ \; & +8w_{r}g_{r}+4w_{r}c_{r}\delta +4w_{r}\delta t \end{array}\right]}{-\delta {\left(\delta -4\right)}^{2}}-kg_{n}^{2}$$

$$\underset{g_{r}}{\max}\pi_{p}^{I}=\frac{{\left(\delta -2t-2w_{r}-2c_{r}+\delta t+2g_{r}+\delta c_{n}-g_{n}\delta \right)}^{2}}{\delta {\left(\delta -4\right)}^{2}}-kg_{r}^{2}$$

The conditions for profit maximization will be satisfied if

$$k>\frac{-4-8\delta -\delta^{2}-\sqrt{16-64\delta +120\delta^{2}+16\delta^{3}+\delta^{4}}}{2(12\delta^{2}-32\delta )}$$

The equilibrium decisions are obtained by solving the system of three equations of the OEM yields.

For the above function, the first derivatives of profit are

$$\begin{array}{l} \frac{\partial \pi_{m}^{I}}{\partial f_{r}}=\frac{\left[\begin{array}{l} 8c_{r}-4\delta t-8\delta -8g_{r}+\delta^{2}c_{n}+\delta^{2}t-4c_{r}\delta \\ +4g_{r}\delta -g_{n}\delta^{2}-6w_{r}\delta +3\delta^{2}+16w_{r}+8t \end{array}\right]}{-\delta {\left(\delta -4\right)}^{2}}\\ \frac{\partial \pi_{m}^{I}}{\partial g_{n}}=\frac{8-8c_{n}+4c_{r}-4t+8g_{n}-4\delta -4g_{r}-2kg_{n}\delta^{2}-32kg_{n}+w_{r}\delta +16kg_{n}\delta }{\delta {\left(\delta -4\right)}^{2}}\\ \frac{\partial \pi_{p}^{I}}{\partial g_{r}}=\frac{\left[\begin{array}{l} 8-8c_{n}+4c_{r}-4t-\delta t+8g_{n}-4\delta -4g_{r}\\ +g_{r}\delta +w_{r}\delta -2kg_{n}\delta^{2}+16kg_{n}\delta -32kg_{n} \end{array}\right]}{{\left(\delta -4\right)}^{2}} \end{array}$$

and the Hessian for the OEM is

$$H=\left(\begin{array}{cc} \frac{2(3\delta -8)}{\delta {\left(\delta -4\right)}^{2}} & \frac{\delta }{{\left(\delta -4\right)}^{2}}\\ \frac{\delta }{{\left(\delta -4\right)}^{2}} & \frac{8-2k\delta^{2}-32k+16k\delta }{{\left(\delta -4\right)}^{2}} \end{array}\right)$$

Based on the above Hessian for the OEM, we can obtain that, for any 1>k>0, 1>δ>0, the profit maximization by OEM will be satisfied. Then, the equilibrium decisions are obtained by solving the system of three equations yields,

$$\begin{array}{c} g_{n}^{I}=\frac{\left[\begin{array}{l} 4c_{n}-4+4t-4k\delta^{2}c_{r}-32k\delta c_{n}+3k\delta^{3}-16k\delta t+\\ 4k\delta^{2}c_{n}+32k\delta -20k\delta^{2}+k\delta^{3}t+k\delta^{3}c_{n}+16k\delta c_{r} \end{array}\right]}{128k^{2}\delta -16k-80k^{2}\delta^{2}+4+4k\delta^{2}+12k^{2}\delta^{3}+k\delta^{3}-32k\delta }\\ g_{r}^{I}=\frac{4(c_{r}+t-\delta +4k\delta c_{n}+4k\delta t-4kc_{r}-4kt)}{128k^{2}\delta -16k-80k^{2}\delta^{2}+4+4k\delta^{2}+12k^{2}\delta^{3}+k\delta^{3}-32k\delta }\\ f_{r}^{I}=\frac{\left[\begin{array}{l} 2k\delta (2\delta^{2}-4-8\delta -2c_{r}\delta -2\delta t+4c_{n}+8c_{r}+12t\\ +3k\delta^{3}-20k\delta^{2}+32k\delta -4k\delta^{2}c_{r}+24k\delta t-4k\delta^{2}c_{n}\\ +k\delta^{3}t+k\delta^{3}c_{n}+24k\delta c_{r}-32kt-8k\delta^{2}t-32kc_{r}) \end{array}\right]}{128k^{2}\delta -16k-80k^{2}\delta^{2}+4+4k\delta^{2}+12k^{2}\delta^{3}+k\delta^{3}-32k\delta } \end{array}$$

Substituting the optimal outcomes of $f_{r}^{I},g_{n}^{I},g_{r}^{I}$ into $q_{n}^{I},q_{r}^{I}$, we can obtain all optimal outcomes of Model I in Table 3.

Note that, based on the outcomes of both models in Table 1, we assume that all parameters and variables in this paper must satisfy non-negativity constraints; that is, we only consider:

$$\frac{\left[\begin{array}{l} 8-32tk-8c_{n}+2k\delta^{3}c_{n}+2k\delta^{3}t\\ -32k\delta^{2}c_{n}-\delta^{3}-6\delta t-8\delta +\delta^{2}t\\ -6k\delta^{3}-64k\delta +40k\delta^{2}-28k\delta^{2}t\\ +6\delta^{2}+96k\delta c_{n}+88k\delta t \end{array}\right]}{6\delta -4k\delta^{2}+8k\delta -8-\delta^{2}+32k}<{\underset{˙}{c}}_{r}=c_{r}<{\dot{c}}_{r}=\frac{4k\delta c_{n}+4k\delta t-\delta +t-4tk}{-1+4k}$$

and

$$k>\underset{˙}{k}=\frac{-4-8\delta -\delta^{2}-\sqrt{16-64\delta +120\delta^{2}+16\delta^{3}+\delta^{4}}}{2(12\delta^{2}-32\delta )}$$

## Appendix B

Proof for Proposition 1. Based on the outcomes in Table 1, then we can get that

$$q_{n}^{C*}-q_{n}^{I*}=\frac{-k\delta^{2}(4k+1)(-4tk+4k\delta c_{n}-4kc_{r}+4k\delta t+c_{r}-\delta +t)}{\left[\begin{array}{l} 128k^{2}\delta -16k-80k^{2}\delta^{2}+4k\delta^{2}\\ +4+12k^{2}\delta^{3}+k\delta^{3}-32k\delta \end{array}\right]\left[\begin{array}{l} k\delta^{2}+12k^{2}\delta^{2}+4k\\ -32k^{2}\delta +8k\delta -1 \end{array}\right]}$$

After simplification, we find that for any ${\underset{˙}{c}}_{r}=c_{r}<{\dot{c}}_{r}$, $k>\underset{˙}{k}$, then $q_{n}^{C*}-q_{n}^{I*}>0$.

Similarly, based on the outcomes in Table 1, then we can get that

$$q_{r}^{C*}-q_{r}^{I*}=\frac{2(-4tk+4k\delta c_{n}-4kc_{r}+4k\delta t+c_{r}-\delta +t)k\delta (4k+1)}{\left[\begin{array}{l} 128k^{2}\delta -16k-80k^{2}\delta^{2}+4k\delta^{2}\\ +4+12k^{2}\delta^{3}+k\delta^{3}-32k\delta \end{array}\right]\left[\begin{array}{l} k\delta^{2}+12k^{2}\delta^{2}+4k\\ -32k^{2}\delta +8k\delta -1 \end{array}\right]}$$

After simplification, we find that for any ${\underset{˙}{c}}_{r}=c_{r}<{\dot{c}}_{r}$, $k>\underset{˙}{k}$, then $q_{r}^{C*}-q_{r}^{I*}<0$.

## Appendix C

Based on the outcomes in Table 1, then we can get that

$$f_{r}^{C*}-f_{r}^{I*}=\frac{\left[\begin{array}{cc} \; & 4c_{r}-4\delta +64k^{2}\delta^{3}-512k^{3}c_{r}\delta -1344k^{4}\delta^{4}t+144k^{4}\delta^{5}t-32kc_{r}\\ \; & +4k^{2}\delta^{4}c_{n}+16k^{2}\delta^{4}t+16k\delta +32k\delta^{2}-\delta^{4}k-1248k^{3}\delta^{3}t\\ \; & -4k^{2}\delta^{4}+48k^{3}\delta^{4}t+24k^{3}\delta^{5}t+k^{2}\delta^{5}t+256k^{3}\delta^{2}c_{r}-16k^{3}\delta^{3}c_{r}\\ \; & +512k^{3}\delta^{2}c_{n}+16k\delta c_{n}-32k\delta c_{r}+48k\delta t-8k^{2}\delta^{3}t-128k^{2}\delta^{2}\\ \; & +2304k^{3}\delta^{2}t-128k^{2}\delta^{2}c_{n}-320k^{2}\delta^{2}t-64k^{2}\delta^{2}c_{r}+256k^{2}\delta c_{r}\\ \; & -320k^{2}\delta t-64k^{2}\delta c_{n}-256k^{3}\delta^{3}c_{n}+16k^{3}\delta^{4}c_{n}+k\delta^{3}c_{r}+64k^{2}c_{r}\\ \; & +4096k^{4}\delta^{3}t+512k^{3}\delta t-4096k^{4}\delta^{2}t \end{array}\right]}{\left[\begin{array}{cc} \; & 128k^{2}\delta -16k-80k^{2}\delta^{2}+4k\delta^{2}\\ \; & +4+12k^{2}\delta^{3}+k\delta^{3}-32k\delta \end{array}\right]\left[\begin{array}{cc} \; & k\delta^{2}+12k^{2}\delta^{2}+4k\\ \; & -32k^{2}\delta +8k\delta -1 \end{array}\right]}$$

After simplification, we find that, there is a threshold of

$$\begin{array}{c} {\bar{c}}_{r}=\frac{\left[\begin{array}{cc} \; & \delta (16k-4+24k^{3}\delta^{4}t+16k^{2}\delta^{3}t+48k^{3}\delta^{3}t-8k^{2}\delta^{2}t\\ \; & -256k^{3}\delta^{2}c_{n}-128k^{2}\delta c_{n}+16k^{3}\delta^{3}c_{n}+4096k^{4}\delta^{2}t\\ \; & +2304k^{3}\delta t+k^{2}\delta^{4}t-320k^{2}\delta t-1248k^{3}\delta^{2}t-k\delta^{3}\\ \; & -1344k^{4}\delta^{3}t+144k^{4}\delta^{4}t-4096k^{4}\delta t+4k^{2}\delta^{3}c_{n}\\ \; & +512k^{3}\delta c_{n}-4k^{2}\delta^{3}+512k^{3}t-64k^{2}c_{n}+48kt\\ \; & -320k^{2}t+16kc_{n}+32k\delta +64k^{2}\delta^{2}-128k^{2}\delta \end{array}\right]}{\left[\begin{array}{cc} \; & 512k^{3}\delta -k\delta^{3}+64k^{2}\delta^{2}-256k^{2}\delta -64k^{2}\\ \; & +32k-256k^{3}\delta^{2}+16k^{3}\delta^{3}+32k\delta -4 \end{array}\right]} \end{array}$$

Below which, $f_{r}^{C}-f_{r}^{I}<0$; otherwise, $f_{r}^{C}-f_{r}^{I}>0$.

## Appendix D

Based on the outcomes in Table 1, then we can get that

$$\pi_{m}^{C*}-\pi_{m}^{I*}=\frac{k{\left[\begin{array}{l} c_{r}-\delta -4tk+4k\delta c_{n}\\ -4kc_{r}+4k\delta t+t \end{array}\right]}^{2}\left[\begin{array}{l} 16-64k-32k^{2}\delta^{3}+8k\delta^{3}-192k^{2}\delta^{2}\\ +512k^{2}\delta +k\delta^{4}-128k\delta +12k^{2}\delta^{4}-16k\delta^{2} \end{array}\right]}{{\left[\begin{array}{l} 128k^{2}\delta -16k-80k^{2}\delta^{2}+4k\delta^{2}\\ +4+12k^{2}\delta^{3}+k\delta^{3}-32k\delta \end{array}\right]}^{2}\left[\begin{array}{l} k\delta^{2}+12k^{2}\delta^{2}+4k\\ -32k^{2}\delta +8k\delta -1 \end{array}\right]}$$

After simplification, we find that for any ${\underset{˙}{c}}_{r}=c_{r}<{\dot{c}}_{r}$, $k>\underset{˙}{k}$, then $\pi_{m}^{C*}-\pi_{m}^{I*}>0$.

## Appendix E

Based on the outcomes in Table 1, then we can get that

$$\pi_{p}^{C*}-\pi_{p}^{I*}=\frac{k\left[\begin{array}{cc} \; & -4{\left(4k\delta t-\delta +4k\delta c_{n}+t+c_{r}-4tk-4kc_{r}\right)}^{2}k(-4-12k^{2}\delta^{4}+k\delta^{3}\\ \; & +32k-64k^{2}-8k^{2}\delta^{3}-16k^{3}\delta^{5}+1024k^{3}\delta -1008k^{3}\delta^{3}+64k\delta \\ \; & +1536k^{3}\delta^{2}-128k^{2}\delta^{2}-512k^{2}\delta -2k^{2}\delta^{5}-4096k^{4}\delta^{2}+96k^{4}\delta^{5}\\ \; & +4096k^{4}\delta^{3}-1216k^{4}\delta^{4}+96k^{3}\delta^{4}) \end{array}\right]}{{\left[\begin{array}{cc} \; & 128k^{2}\delta -16k-80k^{2}\delta^{2}+4k\delta^{2}\\ \; & +4+12k^{2}\delta^{3}+k\delta^{3}-32k\delta \end{array}\right]}^{2}{\left[\begin{array}{cc} \; & k\delta^{2}+12k^{2}\delta^{2}+4k\\ \; & -32k^{2}\delta +8k\delta -1 \end{array}\right]}^{2}}$$

After simplification, we find that for any ${\underset{˙}{c}}_{r}=c_{r}<{\dot{c}}_{r}$, $k>\underset{˙}{k}$, then $\pi_{p}^{C*}-\pi_{p}^{I*}<0$.

## Appendix F

Based on the outcomes in Table 1, then we can get that

$$g_{n}^{C*}-g_{n}^{I*}=\frac{4k\delta^{2}(c_{r}+t-\delta +4k\delta c_{n}+4k\delta t-4kc_{r}-4tk)}{\left[\begin{array}{l} 128k^{2}\delta -16k-80k^{2}\delta^{2}+4k\delta^{2}\\ +4+12k^{2}\delta^{3}+k\delta^{3}-32k\delta \end{array}\right]\left[\begin{array}{l} k\delta^{2}+12k^{2}\delta^{2}+4k\\ -32k^{2}\delta +8k\delta -1 \end{array}\right]}$$

After simplification, we find that for any ${\underset{˙}{c}}_{r}=c_{r}<{\dot{c}}_{r}$, $k>\underset{˙}{k}$, then $g_{n}^{C*}-g_{n}^{I*}>0$.

Based on the outcomes in Table 1, then we can get that

$$g_{r}^{C*}-g_{r}^{I*}=\frac{-(c_{r}+t-\delta +4k\delta c_{n}+4k\delta t-4kc_{r}-4tk)k\delta^{2}(8-32k+12k\delta +\delta )}{\left[\begin{array}{l} 128k^{2}\delta -16k-80k^{2}\delta^{2}+4k\delta^{2}\\ +4+12k^{2}\delta^{3}+k\delta^{3}-32k\delta \end{array}\right]\left[\begin{array}{l} k\delta^{2}+12k^{2}\delta^{2}+4k\\ -32k^{2}\delta +8k\delta -1 \end{array}\right]}$$

After simplification, we find that for any ${\underset{˙}{c}}_{r}=c_{r}<{\dot{c}}_{r}$, $k>\underset{˙}{k}$, then $g_{r}^{C*}-g_{r}^{I*}<0$.

## Appendix G

Based on the outcomes in Table 1, then we can get that

$$G^{C}=g_{n}^{C*}q_{n}^{C*}+g_{r}^{C*}q_{r}^{C*}=\frac{\left[\begin{array}{l} (1-c_{n}-t-8k\delta +k\delta^{2}c_{n}+k\delta^{2}t-4k\delta c_{r}\\ +8k\delta c_{n}+4k\delta t+3k\delta^{2})(6k\delta^{2}-2k\delta^{2}t\\ -2k\delta^{2}c_{n}+\delta^{2}-16k\delta -4k\delta c_{r}+12k\delta t\\ -\delta t-c_{r}\delta +16k\delta c_{n}+2-2c_{n}-2t) \end{array}\right]k}{{\left(k\delta^{2}+12k^{2}\delta^{2}-32k^{2}\delta +8k\delta -1+4k\right)}^{2}};$$

$$\begin{array}{cc} \; & G^{I}=g_{n}^{I*}q_{n}^{I*}+g_{r}^{I*}q_{r}^{I*}=\frac{\left[\begin{array}{cc} \; & (4c_{r}+4t-4\delta +16k\delta c_{n}+16k\delta t-16kc_{r}\\ \; & -16tk)\left(-2\delta +8\right)(4k\delta t-\delta +4k\delta c_{n}+t\\ \; & +c_{r}-4tk-4kc_{r})k \end{array}\right]}{{\left(128k^{2}\delta -16k-80k^{2}\delta^{2}+4+4k\delta^{2}+12k^{2}\delta^{3}+k\delta^{3}-32k\delta \right)}^{2}}+\\ & \frac{\left[\begin{array}{cc} \; & (4c_{n}-4+4t-4k\delta^{2}c_{r}-32k\delta c_{n}+3k\delta^{3}-16k\delta t+4k\delta^{2}c_{n}+32k\delta \\ \; & -20k\delta^{2}+k\delta^{3}t+k\delta^{3}c_{n}+16k\delta c_{r})(-2k\delta^{3}c_{n}+\delta^{3}+6k\delta^{3}-2k\delta^{3}t\\ \; & +24k\delta^{2}c_{n}+20k\delta^{2}t-\delta^{2}t-4\delta^{2}-40k\delta^{2}-\delta^{2}c_{r}-4k\delta^{2}c_{r}+16k\delta c_{r}\\ \; & +4c_{r}\delta +64k\delta -48k\delta t-64k\delta c_{n}+4\delta t+8c_{n}+8t-8)k \end{array}\right]}{{\left(128k^{2}\delta -16k-80k^{2}\delta^{2}+4+4k\delta^{2}+12k^{2}\delta^{3}+k\delta^{3}-32k\delta \right)}^{2}} \end{array}$$

After simplification, we find that, there is a threshold of

$$\Delta c_{r}=\frac{\left[\begin{array}{cc} \; & 8t-64k\delta^{2}c_{n}+256k^{2}\delta^{3}-512tk^{3}+32k\delta +4\delta t+384k^{2}t\\ \; & +20480k^{4}\delta^{3}-20480k^{4}\delta^{4}+6720k^{4}\delta^{5}-96tk-168k^{3}\delta^{6}\\ \; & +4\delta c_{n}-720k^{4}\delta^{6}+512k^{2}\delta^{2}-3072k^{3}\delta^{2}-4608k^{3}\delta^{3}\\ \; & +1904k^{3}\delta^{4}+64k\delta^{2}+544k^{3}\delta^{5}+64k^{2}\delta -32k\delta^{3}+248k^{2}\delta^{4}\\ \; & -44k^{2}\delta^{5}-32k\delta^{2}t-3008k^{3}\delta^{3}t+168k^{3}\delta^{5}t+8k^{2}\delta^{4}c_{n}\\ \; & -32768\delta^{3}c_{n}k^{5}-1024k^{2}\delta^{2}t+32768k^{5}\delta^{2}t-1216k^{4}\delta^{5}c_{n}\\ \; & -1024k^{3}\delta^{3}c_{n}-11904k^{5}\delta^{5}t+3072k^{3}\delta^{2}c_{n}+256k^{2}\delta^{3}c_{n}\\ \; & -464k^{3}\delta^{4}t+240k^{4}\delta^{6}c_{n}-448k^{2}\delta t-k^{2}\delta^{6}c_{n}-2k^{2}\delta^{5}t \end{array}\right]}{\left[\begin{array}{cc} \; & 256k^{3}-10240k^{4}\delta^{3}-84k^{3}\delta^{5}-28k^{2}\delta^{4}-16384k^{5}\delta^{2}+272k^{3}\delta^{4}\\ \; & +128k^{2}\delta^{3}-5376k^{5}\delta^{4}-4+12288k^{4}\delta^{2}+256k^{2}\delta^{2}-240k^{4}\delta^{5}\\ \; & -16k\delta^{2}+2752k^{4}\delta^{4}+16384k^{5}\delta^{3}-5k^{2}\delta^{5}+576k^{5}\delta^{5}-2816k^{3}\delta^{2}\\ \; & +48k-192k^{2}+2k\delta^{3}+992k^{3}\delta^{3} \end{array}\right]}$$

Below which, $G^{C}-G^{I}>0$; otherwise, $G^{C}-G^{I}<0$.
